# The Impact of Influenza Vaccination on Antibiotic Use in the United States, 2010–2017

**DOI:** 10.1093/ofid/ofaa223

**Published:** 2020-06-06

**Authors:** Eili Y Klein, Emily Schueller, Katie K Tseng, Daniel J Morgan, Ramanan Laxminarayan, Arindam Nandi

**Affiliations:** 1 Center for Disease Dynamics, Economics & Policy, Washington, DC, USA; 2 Johns Hopkins University, Baltimore, Maryland, USA; 3 University of Maryland School of Medicine, Baltimore, Maryland, USA; 4 Princeton University, Princeton, New Jersey, USA; 5 University of Washington, Seattle, Washington, USA

**Keywords:** antibiotic consumption, antimicrobial resistance, ecological study, influenza vaccination, upper respiratory tract infections

## Abstract

**Background:**

Influenza, which peaks seasonally, is an important driver for antibiotic prescribing. Although influenza vaccination has been shown to reduce severe illness, evidence of the population-level effects of vaccination coverage on rates of antibiotic prescribing in the United States is lacking.

**Methods:**

We conducted a retrospective analysis of influenza vaccination coverage and antibiotic prescribing rates from 2010 to 2017 across states in the United States, controlling for differences in health infrastructure and yearly vaccine effectiveness. Using data from IQVIA’s Xponent database and the US Centers for Disease Control and Prevention’s FluVaxView, we employed fixed-effects regression analysis to analyze the relationship between influenza vaccine coverage rates and the number of antibiotic prescriptions per 1000 residents from January to March of each year.

**Results:**

We observed that, controlling for socioeconomic differences, access to health care, childcare centers, climate, vaccine effectiveness, and state-level differences, a 10–percentage point increase in the influenza vaccination rate was associated with a 6.5% decrease in antibiotic use, equivalent to 14.2 (95% CI, 6.0–22.4; *P* = .001) fewer antibiotic prescriptions per 1000 individuals. Increased vaccination coverage reduced prescribing rates the most in the pediatric population (0–18 years), by 15.2 (95% CI, 9.0–21.3; *P* < .001) or 6.0%, and the elderly (aged 65+), by 12.8 (95% CI, 6.5–19.2; *P* < .001) or 5.2%.

**Conclusions:**

Increased influenza vaccination uptake at the population level is associated with state-level reductions in antibiotic use. Expanding influenza vaccination could be an important intervention to reduce unnecessary antibiotic prescribing.

At least one-third of all outpatient antibiotic prescriptions nationally are inappropriate [1], and the actual amount may be as high as 76% when considering adherence to guidelines and duration of prescriptions [2]. Antibiotic prescribing peaks during the winter months, traditionally associated with the influenza season [3–5]. Studies have suggested that this pattern in antibiotic prescribing may be driven by a combination of appropriate use (eg, to treat secondary bacterial infections due to influenza) and inappropriate use, including the misprescription of antibiotics to treat viral infections caused by influenza or other viruses (eg, rhinovirus, adenovirus) [6–9]. Thus, reducing influenza cases through increased vaccination could help reduce antibiotic consumption and selection pressure that drives antimicrobial resistance (AMR), which causes >2 million antibiotic-resistant infections and an estimated 35 000–162 000 deaths annually in the United States [10–13].

According to the US Centers for Disease Control and Prevention (CDC), influenza vaccination averted roughly 6.2 million influenza cases and 3.2 million influenza-related medical visits during the 2017–2018 season [14]. For the 2018–2019 season, influenza vaccination coverage in the United States was 62.6% among children (6 months–17 years), 45.3% among adults (18–64 years), and 68.1% among the elderly population (≥65 years) [15]. Meanwhile, rates of outpatient oral antibiotic prescribing in the United States have been found to be highest in pediatric and elderly populations, though patients aged 20–64 account for the greatest number of prescriptions [1].

There is evidence from randomized controlled trials (RCTs) that when the influenza vaccine is well matched to circulating influenza strains, vaccination reduces the proportion of individuals prescribed antibiotics and the number of courses prescribed, compared with placebo or no vaccination [16]. With the exception of 1 study that observed reduced antibiotic use in family and community contacts of vaccinated individuals [17], RCTs on the role of influenza vaccination have measured only the direct impact on patients vaccinated and not the indirect benefits to nonrecipients due to reductions in disease prevalence. Since 2010, the US CDC has recommended annual influenza vaccination for all individuals >6 months of age, an expansion upon its previous recommendation targeting older age groups, based on evidence of the safety and potential health benefits of annual vaccination in all age groups [18]. Though increased vaccination has not been shown to reduce overall visits for influenza-like illness (ILI) among vaccinated individuals due to a high share of noninfluenza viruses in reports of influenza-like illness [19, 20], it has been shown to reduce severe febrile illnesses [19], which should, in turn, reduce antibiotic consumption at the population level. For example, in Ontario, Canada, the introduction of a universal influenza immunization program was associated with a 64% decrease in antibiotic prescribing for influenza-associated respiratory illness compared with other provinces [5]. Evidence on the effect of increased influenza vaccination on antibiotic prescribing in the United States at the population level is limited. The expansion of the annual influenza vaccination recommendation to all persons aged ≥6 months in the United States since 2010 provides an opportunity to address this gap.

## METHODS

We conducted a retrospective observational study to examine the impact of influenza vaccination rates on antibiotic prescribing for the 50 US states and the District of Columbia [21].

### Data and Variables

We obtained state-level monthly data on the number of dispensed antibiotic prescriptions reported by retail pharmacies in the United States from IQVIA’s Xponent database for the years 2009 to 2017. IQVIA (formerly IMS Health) data have been previously used to estimate antibiotic consumption in the United States [3, 22–24]. Antibiotics included in the study are listed in Supplementary Table 1. Seasonal influenza vaccination coverage data were extracted from the CDC FluVaxView database [21] and included the cumulative proportion of the population that had received the influenza vaccine from the August preceding that season through the end of the month under observation. We included state-level monthly vaccination coverage rates for the 2009–2010 to 2016–2017 influenza seasons. Vaccination data were available by age group for pediatric (6 months–17 years), adult (18–64 years), and elderly (≥65 years) populations. Antibiotic prescription rates were similarly available for pediatric (0–18 years), adult (19–64 years), and elderly (≥65 years) populations.

Socioeconomic and structural factors are important drivers of antibiotic prescribing rates [6, 25]. We included the following indicators in our analysis—state-wise poverty rates to control for standard of living [22], the number of dialysis centers per 1 million people to control for general population-level health and health care access [22], and the number of physicians’ offices per 10 000 people to control for overall levels of prescribing [22, 26]. Also included was the number of childcare centers per 10 000 children aged <5 years, as attendance rates of children are known to be positively correlated with antibiotic use [26]. Additionally, we incorporated the difference between mean January and July temperatures to account for seasonal variation [22] and average annual vaccine effectiveness rates to control for the year-to-year effect of vaccination on the burden of influenza. Data on physicians’ offices, dialysis centers, and childcare centers were obtained from the US Census Bureau 2010–2017 County Business Patterns Survey [27]. State-level poverty rates were obtained from the Small Area Income and Poverty Estimates Program of the US Census Bureau [28] and climate data from the National Oceanic and Atmospheric Administration [29]. These covariates were available annually for each state and the District of Columbia. Covariate data for a given year were matched with vaccination and antibiotic data from the influenza season that coincided with January of the same year. The average effectiveness rates of seasonal influenza vaccines, which measure how well matched a vaccine is to circulating strains, were obtained from the CDC [30] and were available annually at the national level. The effectiveness rates did not vary by state and accounted for temporal variation in the fixed-effects model. Data were transformed to rates per 1000 residents using annual state-wise population data from the US Census Bureau (www.census.gov).

### Statistical Analysis

We aggregated antibiotic prescriptions for the months of January through March of each influenza season and used the cumulative influenza vaccination rate from August (the first monthly observation in the FluVaxView data set for each season) to the end of January for each state. We used fixed-effects panel data regression analysis to examine the association between state-level influenza vaccination and antibiotic prescribing rates in the United States, accounting for both spatial and temporal variation in prescribing. This method allowed us to capture variation due to state-specific factors that influence antibiotic consumption, such as varying regulations and norms that govern health care utilization and prescribing practices. We analyzed the impact of vaccination on prescribing by age group, pediatric (0–18 years), adult (19–64 years), and elderly (≥65 years), to account for differences in influenza susceptibility and attack rates by age. Because rates of influenza-like illness are strongly associated with prescribing of certain classes of antibiotics, particularly broad-spectrum penicillins, cephalosporins, macrolides, and fluoroquinolones [31], we evaluated the effect of influenza vaccination on consumption of these antibiotic classes, both overall and for each age group.

## RESULTS

**Figure 1.**
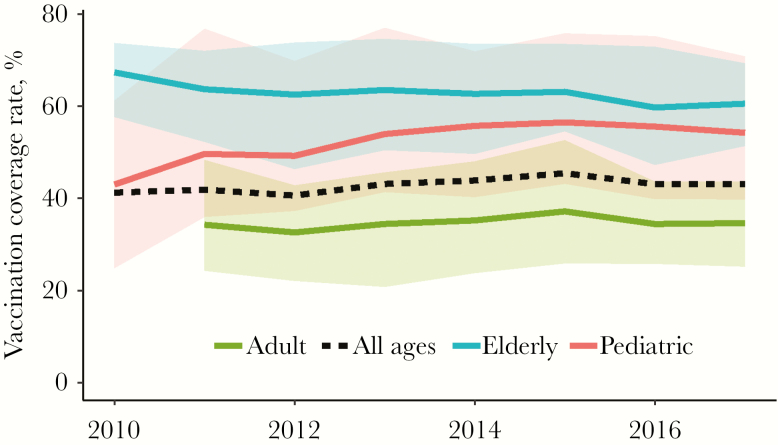
Mean of state influenza vaccination rates (from August to January of the influenza season) from 2010 to 2017 by age group, United States. Each line is the mean value of the state-level vaccination rate of all 50 states and the District of Columbia; the corresponding shaded regions depict the minimum and maximum values for each age group. Source: Centers for Disease Control and Prevention FluVaxView.

**Figure 2.**
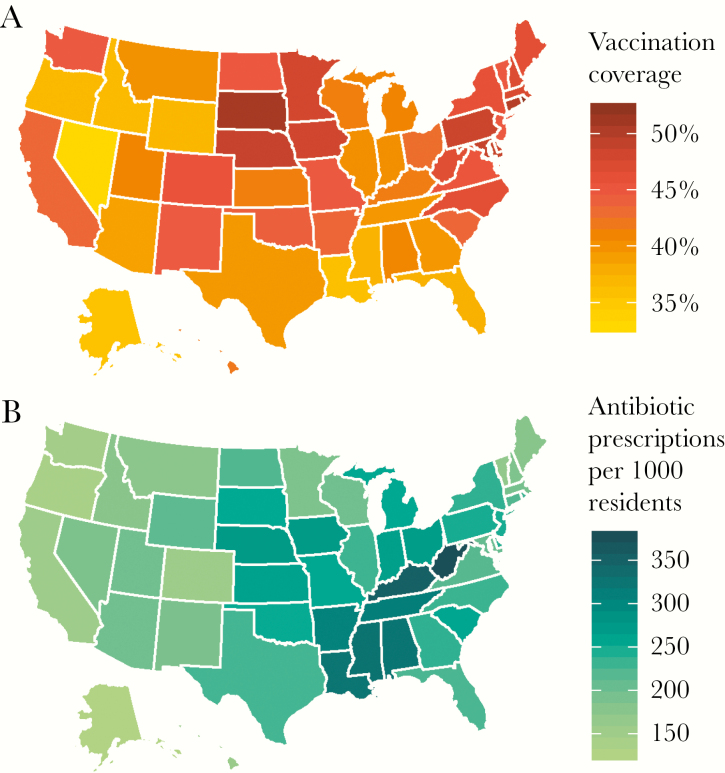
Influenza vaccination coverage and antibiotic consumption rate by state, United States, 2016–2017. A, Cumulative influenza vaccination rate from August to January of the 2016–2017 influenza season. B, Number of antibiotic prescriptions per 1000 residents aggregated for the months of January 2017 to March 2017. Source: Centers for Disease Control and Prevention FluVaxView, IQVIA Xponent, 2000–2015, IQVIA Inc. All rights reserved.

The influenza vaccination coverage rate (measured cumulatively from August to January of the influenza season) ranged across states from 32.8% in Nevada to 52.2% in Rhode Island during the 2016–2017 season (Figures 1 and 2). The mean state-level vaccination rate was 41.2% in 2009–2010 and 43.1% in 2016–2017, while the standard deviation of the vaccination rate ranged from 1.0 in New Mexico to 3.5 in Rhode Island (Supplementary Figures 1 and 2).

**Figure 3.**
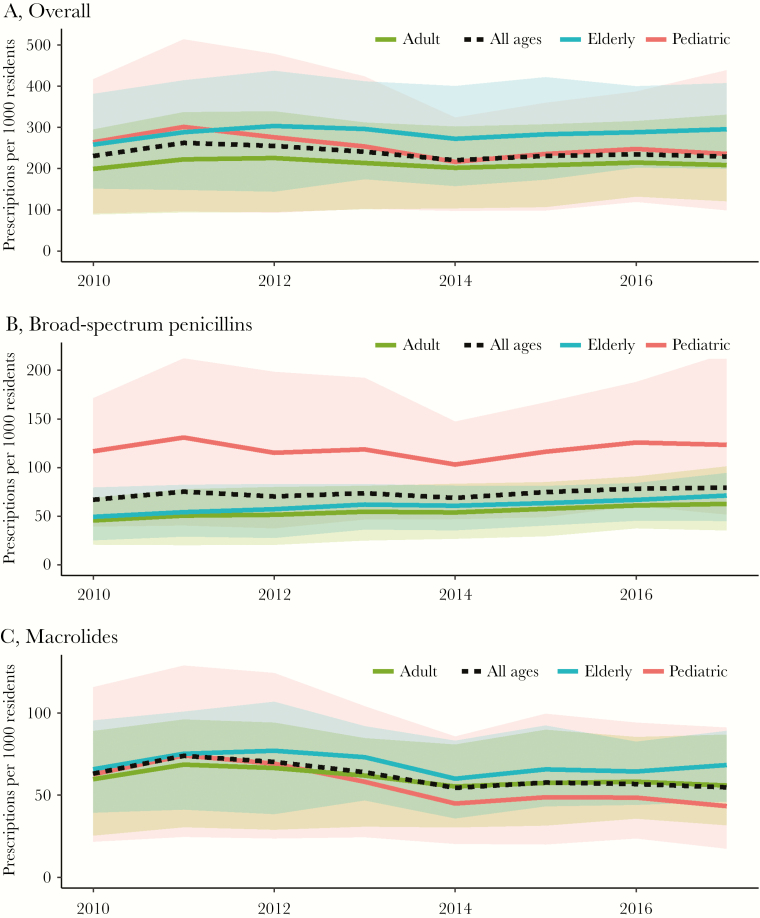
Mean state-level antibiotic consumption rate from 2010 to 2017 by age group, United States. The antibiotic consumption rate was defined as the number of prescriptions per 1000 residents. Each line represents the mean state-level number of antibiotic prescriptions per 1000 residents from January to March across all 50 states and the District of Columbia; the corresponding shaded regions depict the minimum and maximum values for each age group. Overall, the elderly had a slightly higher consumption rate (A), though the pediatric age group had much higher rates of broad-spectrum penicillin consumption (B) and lower rates of macrolide consumption (C). Source: IQVIA Xponent, 2000–2015, IQVIA Inc. All rights reserved.

The mean state consumption rate of all antibiotics from January to March 2017 was 229 prescriptions per 1000 people, ranging from 125 prescriptions per 1000 people in Alaska to 377 prescriptions per 1000 people in West Virginia (Figure 2). In 2010, the mean state antibiotic consumption rate was 231 prescriptions per 1000 people (Figure 3), and the minimum and maximum intrastate variations over time were 20 and 87 prescriptions per 1000 people in Arkansas and Kentucky, respectively (Supplementary Figure 3). The most prescribed drug classes were broad-spectrum penicillins (73 prescriptions per 1000 people from January to March), followed by macrolides (61 prescriptions per 1000 people from January to March), cephalosporins (31 prescriptions per 1000 people from January to March), and fluoroquinolones (27 prescriptions per 1000 people from January to March) (Supplementary Table 2).

We found a significant negative association between increased influenza vaccination coverage rate and antibiotic use rates. A 10–percentage point increase in the influenza vaccination rate was associated with a 6.5% reduction in prescription rates, equivalent to a decrease of 14.2 (95% CI, 6.0–22.4; *P *= .001) prescriptions per 1000 state residents (Table 1), after controlling for covariates. A 10–percentage point increase in vaccine coverage in the pediatric (0–18 years) population was associated with a 6.0% (95% CI, 3.5%–8.5%) reduction in antibiotic prescribing rates among state residents aged 0–18 years, equivalent to a decrease of 15.2 (95% CI, 9.2–21.3; *P* < .001) prescriptions per 1000 state residents. In the elderly (≥65 years) and adult (19–64) populations, prescribing rates were estimated to decrease by 5.2% (95% CI, 2.8%–7.7%) and 4.2% (95% CI, 1.0%–7.4%), respectively, equivalent to a decrease of 12.8 (95% CI, 6.5–19.2; *P* < .001) and 9.0 (95% CI, 2.1–1.59; *P* = .011) prescriptions per 1000 state residents. The percentage of the population below the poverty line was positively associated with increased antibiotic prescribing, while the difference between mean January and July temperatures was negatively associated with prescribing. In the pediatric age group, aged 6 months to 18 years, vaccine effectiveness was positively associated with prescribing after controlling for other factors. Dialysis centers, physician offices, and childcare centers were not significantly associated with prescribing rates.

**Table 1.** Effect of Influenza Vaccination Rate on Antibiotic Prescriptions (Between January and March) per 1000 Residents, United States, 2010–2017

	All Ages *β* (95% CI)	0–18 y, *β* (95% CI)	19–64 y, *β* (95% CI)	≥65 y, *β* (95% CI)
Influenza vaccination coverage, %	–1.42 (–2.24 to –0.60)^**^	–1.52 (–2.13 to –0.90)^***^	–0.90 (–1.59 to –0.21)*	–1.28 (–1.92 to –0.65))^***^
Kidney dialysis centers per 1 million population	0.43 (–0.69 to 1.56)	–0.13 (–1.80 to 1.53)	0.09 (–0.93 to 1.12)	0.43 (–1.13 to 2.00)
Physicians’ offices per 10 000 population	0.24 (–5.86 to 6.35)	6.66 (–11.52 to 24.83)	4.21 (–5.18 to 13.59)	–17.00 (–32.40 to 1.59)*
Childcare centers per 10 000 population aged <5	–0.52 (–1.45 to 0.41)	0.72 (–1.48 to 2.92)	–0.81 (–2.04 to 0.41)	–0.94 (–2.83 to 0.95)
January–July temperature difference	–0.45 (–0.70 to –0.21)^**^	–0.22 (–0.77 to 0.34)	–0.45 (–0.64 to –0.25)^***^	–1.49 (–1.92 to –1.07)^***^
Percentage of population below poverty line	6.63 (4.28 to 8.98)^***^	7.84 (4.05 to 11.63)^***^	2.77 (0.34 to 5.19)*	5.17 (1.99 to 8.35)^**^
Vaccine effectiveness rate	0.16 (–0.003 to 0.31)*	0.46 (0.26 to 0.66)^***^	0.13 (–0.009 to 0.27)	–0.03 (–0.21 to 0.16)

*P < .05; ^**^P < .01; ^***^P < .001.

Influenza vaccination coverage was inversely correlated with antibiotic prescribing for macrolides, tetracyclines, narrow-spectrum penicillins, and aminoglycosides (Table 2; Supplementary Tables 3–10) across all ages. For the pediatric age group, influenza vaccination rates were significantly associated with reductions in all classes except broad-spectrum penicillins and fluoroquinolones. In the elderly population, vaccination was associated with reductions in broad-spectrum penicillins, cephalosporins, and tetracyclines (but not macrolides) and marginally associated with reductions in fluoroquinolones and increases in narrow-spectrum penicillins.

**Table 2.** Effect of Influenza Vaccination Rate on Antibiotic Prescriptions (Between January and March) per 1000 Residents by Antibiotic Class, United States, 2010–2017^a^

	All Ages	0–18 y	19–64 y	≥65 y
	*β* (95% CI)	*β* (95% CI)	*β* (95% CI)	*β* (95% CI)
Broad-spectrum penicillins	–0.02 (–0.31 to 0.28)	–0.27 (–0.56 to 0.03)	0.01 (–0.20 to 0.21)	–0.50 (–0.63 to –0.36)^***^
Macrolides	–0.63 (–0.98 to –0.28)^***^	–0.58 (–0.79 to –0.38)^***^	–0.38 (–0.68 to –0.08)*	–0.12 (–0.35 to 0.11)
Cephalosporins	–0.09 (–0.19 to 0.02)	–0.29 (–0.41 to –0.17)^***^	–0.03 (–0.10 to 0.04)	–0.23 (–0.33 to –0.14)^***^
Fluoroquinolones	–0.08 (–0.21 to 0.06)	–0.03 (–0.08 to 0.03)	–0.05 (–0.18 to 0.08)	–0.20 (–0.39 to –0.01)*
Tetracyclines	–0.19 (–0.26 to –0.11)^***^	–0.06 (–0.08 to –0.03)^***^	–0.14 (–0.24 to –0.05)^**^	–0.16 (–0.23 to –0.10)^***^
Trimethoprim	–0.02 (–0.08 to 0.04)	–0.10 (–0.14 to –0.06)^***^	–0.02 (–0.08 to 0.04)	–0.01 (–0.05 to 0.03)
Narrow-spectrum penicillins	–0.05 (–0.08 to –0.02)^**^	–0.04 (–0.05 to –0.02)^***^	–0.03 (–0.07 to 0.01)	0.02 (0.00 to 0.03)*
Aminoglycosides	–0.17 (–0.23 to –0.10)^***^	–0.14 (–0.18 to –0.09)^***^	–0.08 (–0.13 to –0.04)^***^	0.00 (–0.03 to 0.04)

*P < .05; ^**^P < .01; ^***^P < .001.

^a^Results are coefficients for influenza vaccination coverage controlling for kidney dialysis centers per 1 million population, physicians’ offices per 10 000 population, childcare centers per 10 000 population aged <5, January–July temperature difference, percentage of population below poverty line, and vaccine effectiveness rate.

## DISCUSSION

Annual per capita antibiotic consumption in the United States has decreased in recent years but remains among the highest in the world [32]. In 2017, there were ~821 outpatient antibiotic prescriptions per 1000 people, down slightly from 877 in 2011 [33]. However, a large fraction of antibiotic use in the United States is prescribed inappropriately [1, 2], due in part to lack of awareness of diagnostic guidelines, patient demand, workplace culture, and diagnostic uncertainty [34]. Evidence suggests that vaccination against influenza can reduce antibiotic consumption and inappropriate antibiotic use by (i) reducing the burden of ILI that is commonly mistreated with antibiotics [19, 35], (ii) preventing secondary bacterial infections [36], and (iii) altering patterns of care-seeking behavior and prescribing [37]. Furthermore, reductions in disease prevalence can reduce these burdens even in nonvaccinated individuals, but evidence on this point has been limited. In this study, we found that increases in influenza vaccination were associated with significant reductions in antibiotic prescribing in the United States. These results were strongest for the drugs most likely prescribed for upper respiratory tract infections, including penicillins (eg, amoxicillin), macrolides (eg, azithromycin), and cephalosporins (eg, cephalexin). However, the results were variable by age (Table 2), reflecting how drugs are prescribed differently by age. In addition, reductions were also seen in drugs not as commonly prescribed for upper respiratory infections, such as aminoglycosides, though these are more likely to be prescribed when infections are more severe, which is 1 of the factors vaccines protect against.

Despite efforts to expand influenza immunization, particularly to potential superspreaders such as health care workers [38], many barriers to widespread influenza vaccination remain among both health care workers and the general public [39–41]. Demand-side factors that decrease influenza vaccination uptake include lack of information about the benefits of vaccines [41–44], fear of side effects of vaccines [41, 42], lack of influenza infection in prior seasons [45, 46], and perceived low risk of infection and utility of vaccination [39, 42, 44]. Contextual barriers to influenza vaccination include lack of access to health care facilities [40, 47] and low engagement with health care providers [39, 48]. Individuals who believe their own vaccination status could impact others’ risk of infection have higher uptake rates [41], and health care providers who do not believe that their own vaccination status affects their patients show lower rates [43, 44]. This indicates that achieving and maintaining high enough levels of influenza vaccination to reap the benefits of herd immunity (ie, high enough coverage to limit the spread of disease) necessitates interventions to increase demand for influenza vaccination, particularly among adults who have low engagement with health care providers [39, 48]. Furthermore, electronic communications technology shows some promise in increasing vaccination uptake [39].

This study was an ecological study at the population level, and thus while the results were robust, limitations on the availability of dates to assess potential confounding factors presented some limitations to the analysis. First, while antibiotic prescribing in the United States has been characterized by significant intrastate variation [22], data on influenza vaccination coverage were only available at the state level, which limited our analysis to interstate variation rather than a more granular analysis at the county or health services area level. Second, both vaccination and antibiotic consumption are likely driven by unobserved factors related to overall consumption of health care. We attempted to capture access to health care and general population health by including the number of physicians’ offices per 10 000 population and the number of kidney dialysis centers per 1 million population in our analysis. We included the number of physicians’ offices rather than the number of physicians due to frequency of available data. The number of dialysis centers was weakly correlated with number of physicians’ offices (correlation coefficient, 0.186) but was moderately positively correlated with state-wise poverty rates (correlation coefficient, 0.498). The latter is consistent with research finding that higher poverty levels are associated with poorer public health outcomes in a population [49] and suggests that the number of kidney dialysis centers is an adequate surrogate for general population health. Third, although our models included covariates to capture additional sources of variation in antibiotic prescribing, additional unobserved factors that are associated with vaccination and antibiotic consumption rates, such as unobserved personal beliefs of patients about the value or effectiveness of vaccines, could potentially affect our results. Finally, potential systematic differences in disease transmission or recording of infection could bias the results. For example, influenza seasonality may differ by region in the United States, as cases of ILI tend to peak earlier in the Southeast United States than in the North. However, while in the analysis we examined seasonal antibiotic use by adding prescriptions from January through March of each season and comparing them with vaccination rates at the end of January, varying the time frame of prescription use did not qualitatively change the outcomes.

Our analysis suggests that increasing rates of influenza vaccination coverage may be effective in reducing antibiotic consumption in the United States through reduction of influenza prevalence and limiting secondary bacterial diseases. Substantially boosting seasonal influenza vaccination coverage should be a central element of efforts to reduce use of antibiotics.

## Supplementary Data

Supplementary materials are available at *Open Forum Infectious Diseases* online. Consisting of data provided by the authors to benefit the reader, the posted materials are not copyedited and are the sole responsibility of the authors, so questions or comments should be addressed to the corresponding author.

ofaa223_suppl_Supplementary_Material
