# Supplementary material for: The Impact of Influenza Vaccination on Antibiotic Use in the United States, 2010–2017
Source: Open Forum Infect Dis. 2020 Jun 6;7(7):ofaa223. doi: 10.1093/ofid/ofaa223 (PMC7336555; doi:10.1093/ofid/ofaa223)
Supplement: ofaa223_suppl_Supplementary_Material [file ofaa223_suppl_supplementary_material.docx]

**Supplementary Material**

**for**

**The Impact of Influenza Vaccination on Antibiotic Use in the United States, 2010-2017**

Eili Y. Klein, PhD^1,2*^, Emily Schueller, MA^1^, Katie K. Tseng, MPH^1^, Daniel J Morgan, MD^3^, Ramanan Laxminarayan, PhD^1,4,5^, Arindam Nandi, PhD^1†^

1. Center for Disease Dynamics, Economics & Policy, Washington, DC, USA
2. Johns Hopkins University, Baltimore, MD, USA
3. University of Maryland School of Medicine, Baltimore, MD, USA
4. Princeton University, Princeton, NJ, USA
5. University of Washington, Seattle, WA, USA


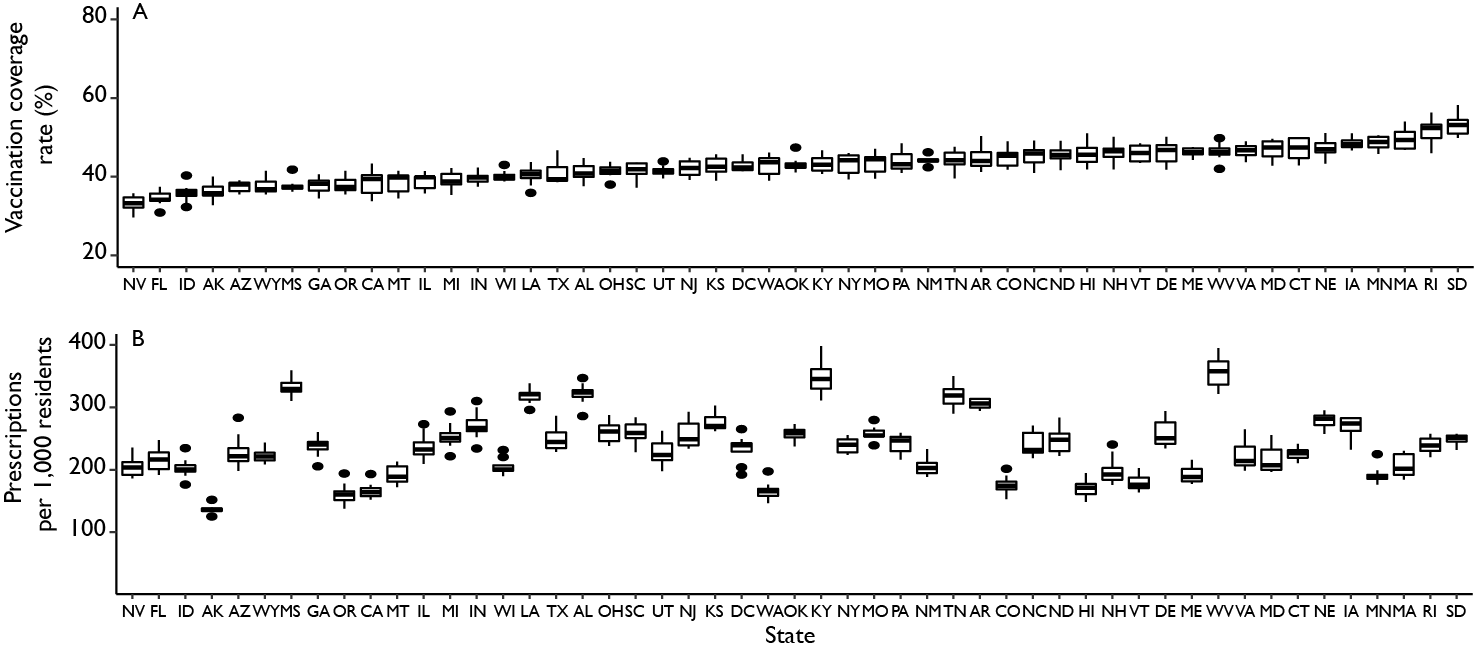


**Figure S1: Influenza vaccination coverage and antibiotic prescription between 2010 and 2017 by state, U.S.** Each bar plots the variability between years for each state and the District of Columbia (A) depicts the cumulative influenza vaccination rates from August to January of each influenza season; (B) depicts the aggregated number of antibiotic prescriptions per 1,000 residents. The line inside the box is the median, the ends of the box are the upper and lower quartiles, and the dots are the extremes. Source: CDC FluVaxView, CDC Outpatient ILI Surveillance Network, IQVIA Xponent, 2000-2015, IQVIA Inc. All rights reserved.

**
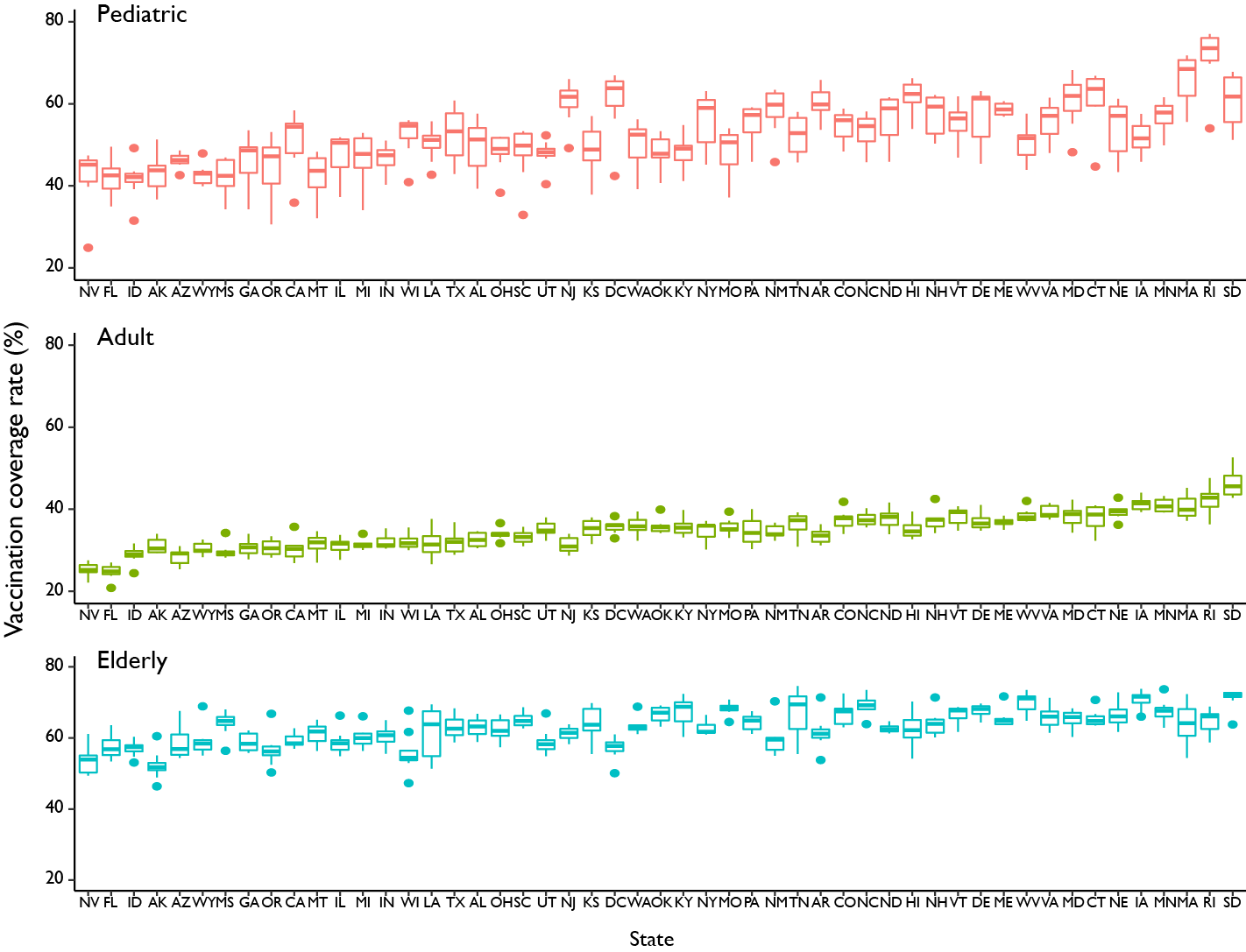
**

Figure S2: Influenza vaccination coverage between 2010-2017 by state and age group, U.S. Each bar plots the variability in the cumulative influenza vaccination rates (from August to January of each influenza season) between years for each state and the District of Columbia by age. The line inside the box is the median, the ends of the box are the upper and lower quartiles, and the dots are the extremes. Source: CDC FluVaxView.

**
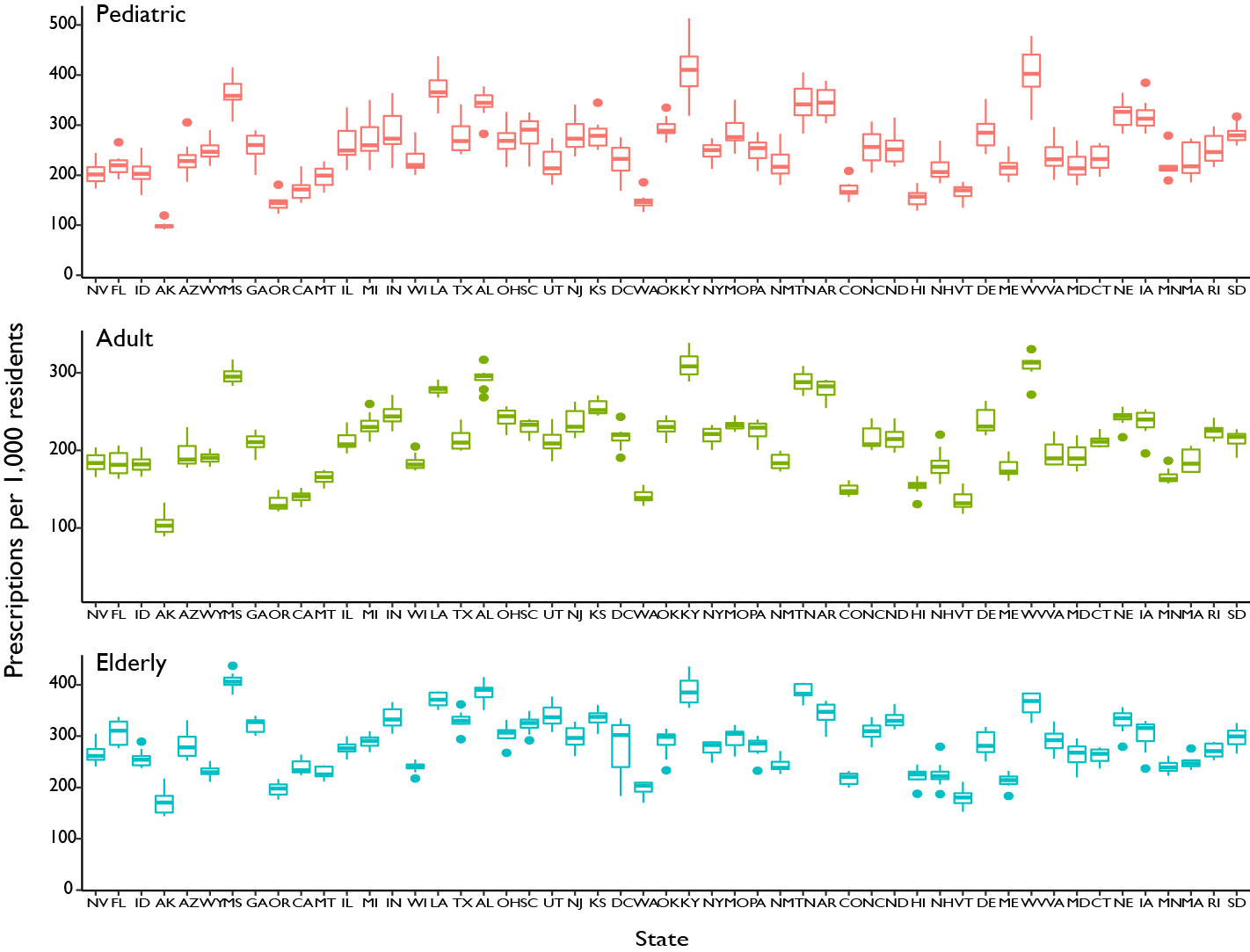
**

Figure S3: Number of antibiotic prescriptions per 1,000 residents between 2010-2017 by state and age group, U.S. Each bar plots the variability in the number of antibiotic prescriptions per 1,000 residents (aggregated from January to March) between years for each state and the District of Columbia. The line inside the box is the median, the ends of the box are the upper and lower quartiles, and the dots are the extremes. Source: IQVIA Xponent, 2000-2015, IQVIA Inc. All rights reserved.

| **Table S1. Antibiotics for which data were available for the years 2009-2017, U.S.** | |
| --- | --- |
| Antibiotic class | Antibiotic |
| Aminoglycosides | Amikacin, atovaquone, gentamicin, kanamycin, neomycin, nitazoxanide, streptomycin, tobramycin |
| Broad-spectrum penicillins | Amoxicillin, amoxicillin-clavulanic acid, ampicillin, ampicillin-sulbactam, piperacillin, piperacillin/tazobactam, sulfadiazine |
| Carbacephems | Loracarbef |
| Carbapenems | Doripenem, ertapenem, imipenem-cilastatin, meropenem |
| Carbenicillins | Carindacillin, ticarcillin |
| Cephalosporins | Cefaclor, cefadroxil, cefalexin, cefazolin, cefdinir, cefditoren pivoxil, cefepime, cefixime, cefotaxime, cefotetan, cefoxitin, cefpodoxime proxetil, cefprozil, cefradine, ceftaroline fosamil, ceftazidime, ceftazidime-avibactam, ceftibuten, ceftizoxime, ceftolozane, ceftriaxone, cefuroxime, cefuroxime axetil, cephalexin, fidaxomicin |
| Chloramphenicols | Chloramphenicol |
| Fluoroquinolones | Ciprofloxacin, gatifloxacin, Gemifloxacin, levofloxacin, lomefloxacin, moxifloxacin, norfloxacin, ofloxacin |
| Glycopeptides | Dalbavancin, oritavancin, telavancin, vancomycin |
| Glycylcycline | Tigecycline |
| Lipopeptides | Daptomycin |
| Macrolides | Azithromycin, clarithromycin, clindamycin, clofazimine, dalfopristin/quinupristin, dirithromycin, erythromycin, lincomycin, telithromycin |
| Monobactams | Aztreonam |
| Narrow-spectrum penicillins | Cloxacillin, dicloxacillin, nafcillin, oxacillin, penicillin G, penicillin V |
| Other | Bacitracin, metronidazole, trimethoprim, trovafloxacin |
| Oxazolidinones | Linezolid, tedizolid |
| Polymyxins | Colistin, polymyxin B, praziquantel |
| Tetracyclines | Demeclocycline, doxycycline, minocycline, oxytetracycline, tetracycline |
| Trimethoprim | Sulfamethoxazole, sulfamethoxazole-trimethoprim, sulfisoxazole |
|  |  |

| Table S2. Mean number of antibiotic prescriptions (between January and March) per 1,000 residents by class, U.S. 2010-2017 | | | | |
| --- | --- | --- | --- | --- |
|  | All Ages | 0–18 years | 19–64 years | ≥65 years |
|  | N = 233 | N = 248 | N = 206 | N = 289 |
| Broad-spectrum penicillins | 73 (31.2) | 118 (47.5) | 54 (26) | 61 (21.2) |
| Macrolides | 61 (26.1) | 55 (22) | 59 (28.7) | 71 (24.6) |
| Cephalosporins | 31 (13.3) | 43 (17.3) | 23 (11.2) | 39 (13.6) |
| Fluoroquinolones | 27 (11.7) | 3 (1.3) | 28 (13.5) | 65 (22.4) |
| Tetracyclines | 17 (7.3) | 12 (4.7) | 18 (8.6) | 21 (7.4) |
| Trimethoprim | 16 (6.7) | 12 (4.9) | 15 (7.4) | 22 (7.5) |
| Narrow-spectrum penicillins | 6 (2.4) | 4 (1.5) | 6 (3.1) | 5 (1.6) |
| Aminoglycosides | 1 (0.5) | 2 (0.6) | 1 (0.4) | 1 (0.5) |
| Other**^a^** | 2 (0.9) | 1 (0.2) | 2 (1) | 4 (1.3) |
| Note: Parentheses is percentage of total antibiotic prescriptions | | | | |
| **^a^** Other class includes glycopeptides, oxazolidinones, polymyxins, carbapenems, lipopeptides, chloramphenicols, monobactams, carbenicillins, and unidentified. | | | | |

| Table S3. Impact of vaccination rate on broad-spectrum penicillin prescriptions (between January and March) per 1,000 residents, U.S. 2010-2017 | | | | |
| --- | --- | --- | --- | --- |
|  | All Ages | 0–18 years | 19–64 years | ≥65 years |
|  | 𝛽 (95% CI) | 𝛽 (95% CI) | 𝛽 (95% CI) | 𝛽 (95% CI) |
| Influenza vaccination coverage (%) | -0.02 (-0.31-0.28) | -0.27 (-0.56-0.03) | 0.01 (-0.20-0.21) | -0.50 (-0.63--0.36)*** |
| Kidney dialysis centers per 1 million population | 0.84 (0.38-1.30)*** | 0.92 (-0.02-1.87) | 0.73 (0.38-1.07)*** | 0.68 (0.26-1.10)** |
| Physicians' offices per 10,000 population | -4.81 (-7.97--1.65)** | -2.76 (-10.62-5.09) | -5.16 (-8.54--1.79)** | -9.25 (-13.23--5.26)*** |
| Childcare centers per 10,000 population under five | -0.35 (-0.70-0.01) | 0.06 (-0.65-0.78) | -0.41 (-0.84-0.02) | -0.33 (-0.74-0.08) |
| January-July temperature difference | -0.17 (-0.26--0.08)*** | -0.17 (-0.42-0.08) | -0.16 (-0.23--0.09)*** | -0.35 (-0.46--0.23)*** |
| Percentage of population below poverty line | -0.60 (-1.60-0.40) | -0.39 (-2.50-1.72) | -1.98 (-2.70--1.26)*** | -1.54 (-2.39--0.69)*** |
| Vaccine effectiveness rate | 0.04 (-0.01-0.10) | 0.20 (0.10-0.30)*** | 0.00 (-0.04-0.05) | -0.06 (-0.11-0.00) |
| Note: CI = confidence interval; * p<0.05, **p<0.01, ***p<0.001 | | | | |

| Table S4. Impact of vaccination rate on macrolide prescriptions (between January and March) per 1,000 residents, U.S. 2010-2017 | | | | |
| --- | --- | --- | --- | --- |
|  | All Ages | 0–18 years | 19–64 years | ≥65 years |
|  | 𝛽 (95% CI) | 𝛽 (95% CI) | 𝛽 (95% CI) | 𝛽 (95% CI) |
| Influenza vaccination coverage (%) | -0.63 (-0.98--0.28)*** | -0.58 (-0.79--0.38)*** | -0.38 (-0.68--0.08)* | -0.12 (-0.35-0.11) |
| Kidney dialysis centers per 1 million population | -0.32 (-0.69-0.06) | -0.65 (-1.16--0.15)* | -0.28 (-0.69-0.14) | -0.29 (-0.76-0.18) |
| Physicians' offices per 10,000 population | 5.04 (2.49-7.59)*** | 6.91 (1.40-12.41)* | 7.22 (4.04-10.40)*** | 0.97 (-4.15-6.09) |
| Childcare centers per 10,000 population under five | -0.12 (-0.56-0.32) | 0.24 (-0.66-1.13) | -0.27 (-0.68-0.14) | 0.00 (-0.63-0.64) |
| January-July temperature difference | -0.07 (-0.17-0.03) | -0.06 (-0.25-0.13) | -0.09 (-0.17-0.00)* | -0.33 (-0.48--0.18)*** |
| Percentage of population below poverty line | 2.99 (2.23-3.75)*** | 4.44 (3.43-5.45)*** | 1.48 (0.58-2.38)** | 2.22 (1.24-3.19)*** |
| Vaccine effectiveness rate | 0.06 (0.01-0.11)* | 0.11 (0.05-0.18)*** | 0.08 (0.02-0.13)** | 0.03 (-0.03-0.08) |
| Note: CI = confidence interval; * p<0.05, **p<0.01, ***p<0.001 | | | | |

| Table S5. Impact of vaccination rate on cephalosporin prescriptions (between January and March) per 1,000 residents, U.S. 2010-2017 | | | | | |
| --- | --- | --- | --- | --- | --- |
|  | All Ages | 0–18 years | 19–64 years | ≥65 years |  |
|  | 𝛽 (95% CI) | 𝛽 (95% CI) | 𝛽 (95% CI) | 𝛽 (95% CI) |  |
| Influenza vaccination coverage (%) | -0.09 (-0.19-0.02) | -0.29 (-0.41--0.17)*** | -0.03 (-0.10-0.04) | -0.23 (-0.33--0.14)*** |  |
| Kidney dialysis centers per 1 million population | 0.13 (-0.04-0.31) | 0.05 (-0.27-0.37) | 0.10 (-0.02-0.23) | 0.31 (0.06-0.57)* |  |
| Physicians' offices per 10,000 population | -0.85 (-2.03-0.32) | 1.25 (-2.14-4.63) | -1.41 (-2.72--0.11)* | -4.66 (-7.02--2.30)*** |  |
| Childcare centers per 10,000 population under five | 0.02 (-0.13-0.17) | 0.37 (-0.09-0.83) | -0.08 (-0.24-0.09) | -0.18 (-0.44-0.07) |  |
| January-July temperature difference | -0.08 (-0.11--0.04)*** | -0.02 (-0.11-0.07) | -0.08 (-0.11--0.05)*** | -0.26 (-0.34--0.18)*** |  |
| Percentage of population below poverty line | 0.48 (0.06-0.91)* | 0.83 (0.01-1.65)* | 0.22 (-0.11-0.56) | -0.45 (-0.97-0.07) |  |
| Vaccine effectiveness rate | 0.03 (0.01-0.05)** | 0.08 (0.05-0.11)*** | 0.01 (0.00-0.03) | -0.01 (-0.04-0.02) |  |
| Note: CI = confidence interval; * p<0.05, **p<0.01, ***p<0.001 | | | | | |

| Table S6. Impact of vaccination rate on fluoroquinolone prescriptions (between January and March) per 1,000 residents, U.S. 2010-2017 | | | | |
| --- | --- | --- | --- | --- |
|  | All Ages | 0–18 years | 19–64 years | ≥65 years |
|  | 𝛽 (95% CI) | 𝛽 (95% CI) | 𝛽 (95% CI) | 𝛽 (95% CI) |
| Influenza vaccination coverage (%) | -0.08 (-0.21-0.06) | -0.03 (-0.08-0.03) | -0.05 (-0.18-0.08) | -0.20 (-0.39--0.01)* |
| Kidney dialysis centers per 1 million population | -0.12 (-0.29-0.05) | -0.07 (-0.14-0.00) | -0.29 (-0.52--0.06)* | -0.38 (-0.80-0.04) |
| Physicians' offices per 10,000 population | 0.95 (-0.13-2.03) | 0.78 (-0.44-2.00) | 2.76 (0.81-4.72)** | -0.29 (-3.26-2.69) |
| Childcare centers per 10,000 population under five | -0.20 (-0.37--0.03)* | 0.04 (-0.04-0.13) | -0.31 (-0.58--0.05)* | -0.49 (-0.94--0.04)* |
| January-July temperature difference | -0.03 (-0.07-0.01) | 0.01 (-0.02-0.05) | -0.02 (-0.07-0.03) | -0.26 (-0.35--0.17)*** |
| Percentage of population below poverty line | 1.77 (1.45-2.09)*** | 0.90 (0.68-1.12)*** | 1.31 (0.83-1.78)*** | 4.07 (3.28-4.85)*** |
| Vaccine effectiveness rate | -0.03 (-0.05--0.01)** | 0.01 (0.00-0.02)** | -0.02 (-0.04-0.01) | -0.08 (-0.12--0.03)** |
| Note: CI = confidence interval; * p<0.05, **p<0.01, ***p<0.001 | | | | |

| Table S7. Impact of vaccination rate on tetracycline prescriptions (between January and March) per 1,000 residents, U.S. 2010-2017 | | | | |
| --- | --- | --- | --- | --- |
|  | All Ages | 0–18 years | 19–64 years | ≥65 years |
|  | 𝛽 (95% CI) | 𝛽 (95% CI) | 𝛽 (95% CI) | 𝛽 (95% CI) |
| Influenza vaccination coverage (%) | -0.19 (-0.26--0.11)*** | -0.06 (-0.08--0.03)*** | -0.14 (-0.24--0.05)** | -0.16 (-0.23--0.10)*** |
| Kidney dialysis centers per 1 million population | 0.08 (-0.03-0.18) | -0.10 (-0.19--0.01)* | 0.10 (-0.03-0.22) | 0.29 (0.13-0.46)*** |
| Physicians' offices per 10,000 population | -0.99 (-1.76--0.22)* | -0.26 (-1.56-1.04) | -1.35 (-2.73-0.03) | -3.26 (-5.02--1.51)*** |
| Childcare centers per 10,000 population under five | 0.13 (0.03-0.22)** | -0.04 (-0.18-0.11) | 0.21 (0.05-0.38)* | 0.09 (-0.10-0.28) |
| January-July temperature difference | -0.08 (-0.11--0.05)*** | 0.00 (-0.02-0.02) | -0.09 (-0.13--0.05)*** | -0.18 (-0.24--0.12)*** |
| Percentage of population below poverty line | -0.09 (-0.29-0.11) | 0.28 (0.13-0.43)*** | -0.08 (-0.35-0.19) | -1.04 (-1.37--0.71)*** |
| Vaccine effectiveness rate | 0.02 (0.00-0.03)* | 0.01 (0.00-0.02)** | 0.02 (0.01-0.04)** | 0.02 (0.00-0.05)* |
| Note: CI = confidence interval; * p<0.05, **p<0.01, ***p<0.001 | | | | |

| Table S8. Impact of vaccination rate on trimethoprim prescriptions (between January and March) per 1,000 residents, U.S. 2010-2017 | | | | | |
| --- | --- | --- | --- | --- | --- |
|  | All Ages | 0–18 years | 19–64 years | ≥65 years | |
|  | 𝛽 (95% CI) | 𝛽 (95% CI) | 𝛽 (95% CI) | 𝛽 (95% CI) | |
| Influenza vaccination coverage (%) | -0.02 (-0.08-0.04) | -0.10 (-0.14--0.06)*** | -0.02 (-0.08-0.04) | -0.01 (-0.05-0.03) | |
| Kidney dialysis centers per 1 million population | -0.08 (-0.17-0.01) | -0.21 (-0.34--0.08)** | -0.05 (-0.12-0.03) | -0.08 (-0.22-0.06) | |
| Physicians' offices per 10,000 population | -0.44 (-1.12-0.23) | -0.18 (-1.53-1.16) | -0.89 (-1.67--0.10)* | -1.67 (-3.20--0.13)* | |
| Childcare centers per 10,000 population under five | 0.01 (-0.07-0.09) | 0.06 (-0.12-0.25) | -0.02 (-0.12-0.08) | 0.00 (-0.19-0.18) | |
| January-July temperature difference | -0.06 (-0.08--0.03)*** | -0.02 (-0.04-0.01) | -0.06 (-0.08--0.04)*** | -0.12 (-0.15--0.08)*** | |
| Percentage of population below poverty line | 0.48 (0.32-0.63)*** | 0.78 (0.55-1.02)*** | 0.27 (0.13-0.42)*** | 0.18 (-0.05-0.41) | |
| Vaccine effectiveness rate | 0.00 (-0.01-0.01) | 0.01 (0.00-0.02) | -0.01 (-0.02-0.00) | 0.01 (-0.01-0.02) | |
| Note: CI = confidence interval; * p<0.05, **p<0.01, ***p<0.001 | | | | |  |

| Table S9. Impact of vaccination rate on narrow-spectrum penicillin prescriptions (between January and March) per 1,000 residents, U.S. 2010-2017 | | | | |  |
| --- | --- | --- | --- | --- | --- |
|  | All Ages | 0-18 years old | 19-64 years old | 65+ years old | |
|  | 𝛽 (95% CI) | 𝛽 (95% CI) | 𝛽 (95% CI) | 𝛽 (95% CI) | |
| Influenza vaccination coverage (%) | -0.05 (-0.08--0.02)** | -0.04 (-0.05--0.02)*** | -0.03 (-0.07-0.01) | 0.02 (0.00-0.03)* | |
| Kidney dialysis centers per 1 million population | -0.07 (-0.12--0.02)* | -0.06 (-0.10--0.01)* | -0.06 (-0.11-0.00)* | -0.03 (-0.07-0.01) | |
| Physicians' offices per 10,000 population | 1.10 (0.66-1.54)*** | 0.55 (0.23-0.86)*** | 1.01 (0.54-1.48)*** | 0.53 (0.25-0.81)*** | |
| Childcare centers per 10,000 population under five | -0.01 (-0.06-0.04) | 0.00 (-0.04-0.04) | 0.00 (-0.05-0.05) | -0.01 (-0.04-0.02) | |
| January-July temperature difference | 0.00 (-0.01-0.01) | 0.01 (0.00-0.02) | -0.01 (-0.02-0.01) | -0.02 (-0.03--0.01)*** | |
| Percentage of population below poverty line | 0.23 (0.13-0.34)*** | 0.02 (-0.07-0.12) | 0.31 (0.20-0.43)*** | 0.19 (0.09-0.29)*** | |
| Vaccine effectiveness rate | 0.01 (0.01-0.02)*** | 0.01 (0.01-0.02)*** | 0.01 (0.00-0.02)*** | 0.01 (0.01-0.02)*** | |
| Note: CI = confidence interval; * p<0.05, **p<0.01, ***p<0.001 | | | | |  |

| Table S10. Impact of vaccination rate on aminoglycoside prescriptions (between January and March) per 1,000 residents, U.S. 2010-2017 | | | | |
| --- | --- | --- | --- | --- |
|  | All Ages | 0-18 years old | 19-64 years old | 65+ years old |
|  | 𝛽 (95% CI) | 𝛽 (95% CI) | 𝛽 (95% CI) | 𝛽 (95% CI) |
| Influenza vaccination coverage (%) | -0.17 (-0.23--0.10)*** | -0.14 (-0.18--0.09)*** | -0.08 (-0.13--0.04)*** | 0.00 (-0.03-0.04) |
| Kidney dialysis centers per 1 million population | -0.03 (-0.08-0.02) | -0.02 (-0.10-0.06) | -0.04 (-0.09-0.01) | -0.03 (-0.08-0.02) |
| Physicians' offices per 10,000 population | 0.42 (-0.09-0.92) | 0.35 (-0.58-1.27) | 0.60 (0.01-1.19)* | 0.57 (0.00-1.14)* |
| Childcare centers per 10,000 population under five | 0.01 (-0.07-0.08) | -0.01 (-0.15-0.13) | 0.02 (-0.04-0.09) | 0.03 (-0.04-0.10) |
| January-July temperature difference | 0.02 (0.00-0.04) | 0.02 (-0.01-0.05) | 0.02 (0.00-0.03) | 0.02 (0.00-0.04) |
| Percentage of population below poverty line | 0.44 (0.32-0.55)*** | 0.74 (0.55-0.94)*** | 0.26 (0.16-0.35)*** | 0.43 (0.31-0.54)*** |
| Vaccine effectiveness rate | 0.01 (0.00-0.02)* | 0.02 (0.01-0.03)*** | 0.01 (0.00-0.01)** | 0.02 (0.01-0.03)*** |
| Note: CI = confidence interval; * p<0.05, **p<0.01, ***p<0.001 | | | |  |
